# The acute effects of high-intensity jack-knife stretching on the flexibility of the hamstrings

**DOI:** 10.1038/s41598-021-91645-x

**Published:** 2021-06-09

**Authors:** Kosuke Takeuchi, Kazunori Akizuki, Masatoshi Nakamura

**Affiliations:** 1 Department of Physical Therapy, Kobe International University, Kobe, Hyogo Japan; 2 Institute for Human Movement and Medical Sciences, Niigata University of Health and Welfare, Niigata, Niigata Japan

**Keywords:** Preventive medicine, Rehabilitation

## Abstract

The purpose of the present study was to examine the acute effects of high-intensity jack-knife stretching for 60 s on flexibility of the hamstrings. Twelve healthy participants underwent jack-knife stretching for 60 s (3 repetitions of 20 s stretching with 30 s intervals) at two different intensities based on the point of discomfort (POD and PODmax). To examine any change in flexibility, knee extension range of motion (ROM), passive torque at end ROM, and muscle–tendon unit stiffness were measured before and after stretching. To evaluate hamstrings pain, a numerical rating scale (NRS) was described. The knee extension ROM (p < 0.01) and passive torque at end ROM (p < 0.05) were significantly increased at both intensities. The muscle–tendon unit stiffness was significantly decreased in PODmax intensity (p < 0.01), but there was no change in POD intensity (p = 0.18). The median values of NRS during the stretching were 0 and 6–7 in POD and PODmax intensity, respectively, although it was 0 immediately after the stretching protocol in both intensities. These data suggested that high-intensity jack-knife stretching is an effective and safe method to decrease muscle–tendon unit stiffness of the hamstrings.

## Introduction

Muscle strain is a frequent sports injury and occurs most often in the hamstrings^1–4^. To prevent muscle strain, it is important to decrease the muscle–tendon unit stiffness of the hamstrings^5–7^. When the muscle–tendon unit stiffness is high, the demands in energy absorption and release may rapidly exceed the capacity of the muscle–tendon unit, which may cause a higher risk of injuries^8,9^. Static^10,11^ and dynamic^12^ stretching effectively decreases the muscle–tendon unit stiffness of the hamstrings. Therefore, a recent review study recommends using stretching exercises as a fundamental warm-up component before recreational sport participation due to its potential positive effect on decrement in the muscle–tendon unit stiffness and musculotendinous injury prevention^13^.

Recently, high-intensity static stretching has been reported as a method to effectively decrease the muscle–tendon unit stiffness^14–16^. The intensity of static stretching is determined according to the range of motion (ROM)^14,17,18^ or point of discomfort (POD)^15,16^ of each participant. Previous studies performed high-intensity static stretching at the intensity of 120%ROM^14^ and 120%POD or more^15,16^ and reported that high-intensity static stretching effectively decreased the muscle–tendon unit stiffness of the hamstrings even if with a short duration of high-intensity stretching (≤ 20 s). However, high-intensity static stretching is associated with severe pain^14–16,18^, although safety at that intensity has been reported^15,16^. In previous studies, high-intensity static stretching was passively performed^14–16,18^. Therefore, it remains unclear whether high-intensity static stretching, which is accompanied by severe pain, can be performed effectively and safely by oneself. It is necessary to develop an effective and safe method of high-intensity self-stretching in order to use it in sports.

Jack-knife stretching is an effective self-stretching technique for the hamstrings^19–21^. It uses a combination of static and dynamic stretching^20^ and can be performed alone without using any equipment^19–21^. Previous studies reported that jack-knife stretching effectively increased the ROM of the hamstrings in healthy adults^20^ and soccer players^19^. To our best knowledge, the acute effect of jack-knife stretching on the muscle–tendon unit stiffness of the hamstrings has not been examined. However, it may be possible to develop a self-stretching technique that can effectively decrease the muscle–tendon unit stiffness of the hamstrings by performing jack-knife stretching at high-intensity.

Therefore, the purpose of the present study was to compare the acute effects of different intensities (POD and PODmax) of jack-knife stretching on the flexibility of the hamstrings. Because the effect of jack-knife stretching on the muscle–tendon unit stiffness is unknown, the effects of normal (POD) and high-intensity (PODmax) jack-knife stretching were compared in this study. The hypothesis of the study was that high-intensity jack-knife stretching effectively decreases the muscle–tendon unit stiffness of the hamstrings, based on previous studies^14–16^.

## Methods

### Participants

**Figure 1:**
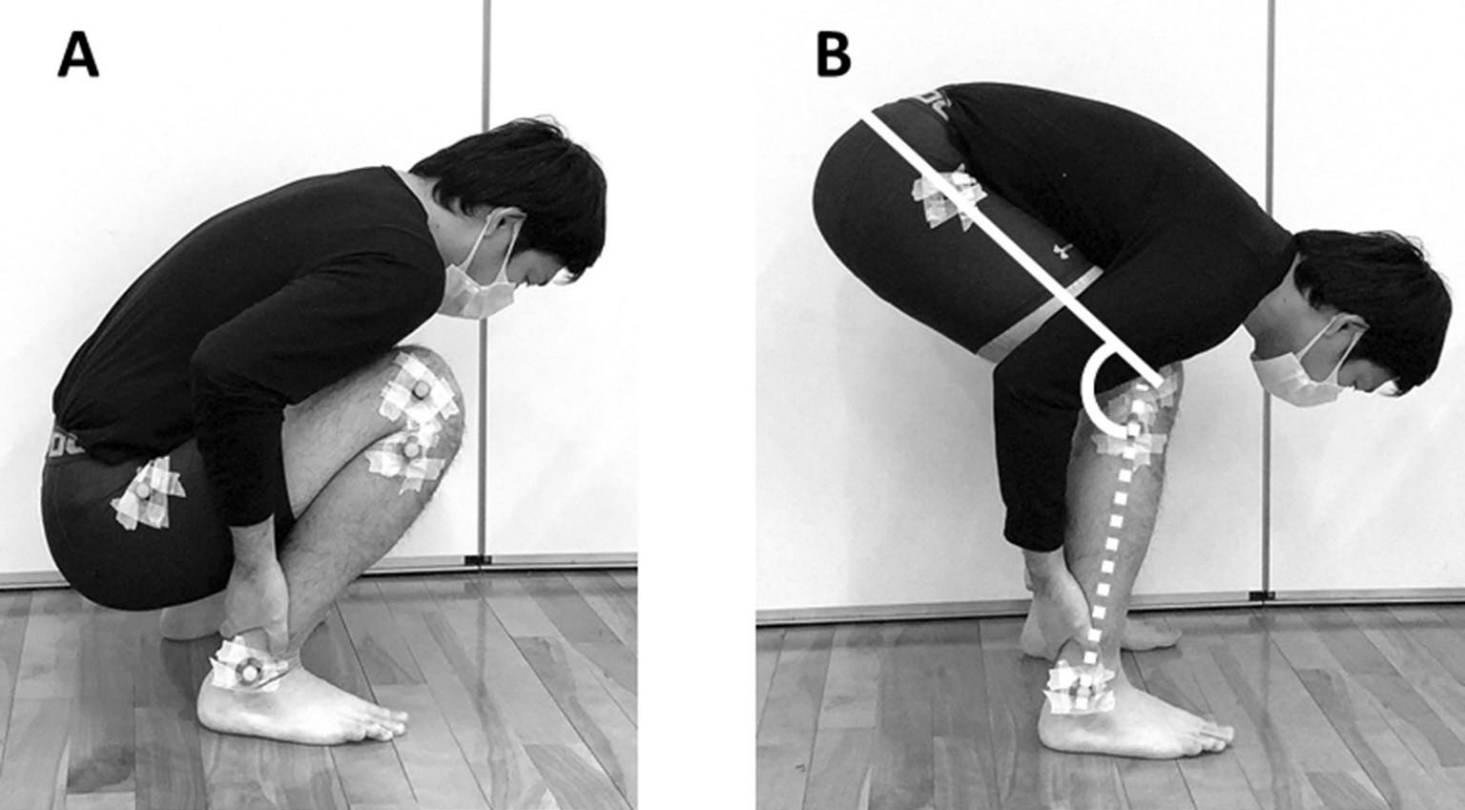
Posture of jack-knife stretching. (**A**) Starting position, (**B**) Stretching position. Reflective markers were placed on the right greater trochanter, lateral condyle of femur, fibular head, and lateral malleolus. The knee angle during jack-knife stretching was defined as the angle between the femur (solid white line) and the fibula (dashed white line).

Nine healthy men (20.9 ± 0.3 years, 169.8 ± 6.8 cm, 59.9 ± 4.1 kg) and three healthy women (20.7 ± 0.6 years, 154.3 ± 4.0 cm, 46.7 ± 5.7 kg) were recruited. Participants who regularly performed any flexibility and strength training or who had a history of lower limb pathology were excluded. Previous studies that examined the effects of high-intensity static stretching of the hamstrings reported large effect sizes for the muscle–tendon unit stiffness^15,16^. Therefore, the sample size of the muscle–tendon unit stiffness was calculated with a power of 95%, alpha error of 0.05, and effect size f of 0.40 (large) using G*Power 3.1 software (Heinrich Heine University, Düsseldorf, Germany), and the results showed that the requisite number of participants for this study was 12. All participants were informed of the requirements and risks associated with their involvement in this study and signed a written informed consent document. The study was performed in accordance with the Declaration of Helsinki (1964). Written informed consent for publication of an image of Fig. 1 was obtained from the participant. The Ethics Committee of Kobe International University approved the study (Procedure #G2020-159).

### Procedure

The participants underwent two different intensities of jack-knife stretching, in random order. They visited two times on separate days, with an interval of 1 week between visits. To assess changes in the flexibility of the right hamstrings (dominant limb of all participants), knee extension ROM, passive torque at end ROM, and muscle–tendon unit stiffness were measured before and immediately after jack-knife stretching. The dominant leg was determined by asking the participant’s preferred leg when kicking a ball^18^. The flexibility measurements were performed without any warm-up. In addition, to investigate the pain in the right hamstrings, a numerical rating scale (NRS) was examined during jack-knife stretching (Repetitions 1, 2, and 3, respectively) and immediately after the stretching protocol (post measurement). The experiment was performed in a university laboratory, where the temperature was maintained at 25 °C.

### Flexibility assessment

The flexibility assessment was performed in the same fashion as previous studies^14–16^. A previous study reported that the reliability of the measurements used in this study was acceptable^14^. An isokinetic dynamometer machine (CYBEX NORM, Humac, California, USA) was used in the present study. This study used a sitting position with the hip joint flexed, which has been shown to efficiently stretch the hamstrings^14^. The participants were seated on a chair with the seat tilted maximally, and a wedge-shaped cushion was inserted between the trunk and the backrest, which set the angle between the seat and the back at approximately 60°. A previous study, which used the same assessment, reported that the average angle of hip flexion was 111.2° ± 2.5°^14,16^. The chest, pelvis, and right thigh were stabilized with straps. The right knee joint was aligned with the axis of the rotation of the isokinetic dynamometer machine. The lever arm attachment was placed just proximal to the malleolus medialis and stabilized with straps. In the present study, reported knee angles were measured using the isokinetic dynamometer machine. A 90° angle between the lever arm and floor was defined as 0° of knee flexion/extension. The participants were instructed to relax during the flexibility assessment.

### Knee extension ROM

The knee joint was passively extended from 0° to the POD at 5°/s. A previous study showed that this velocity does not cause stretch reflex^22^. ROM was defined as the range from 0° to the maximum knee extension angle.

### Passive torque at end ROM

The passive torque during ROM measurement was recorded in the isokinetic dynamometer machine. The passive torque at end ROM was used for further analyses.

### Calculation of muscle–tendon unit stiffness

Calculation of the muscle–tendon unit stiffness of the hamstrings was performed in the same fashion as previous studies^14,16,23,24^. The muscle–tendon unit stiffness was defined as the value of the slope of the regression line that was calculated from the torque–angle relationship using the least-squares method^14,16,23,24^. The muscle–tendon unit stiffness was calculated from the same knee extension angle range before and after jack-knife stretching. The calculated knee extension angle range was defined as the angle from the 50% maximum knee extension angle to the maximum knee extension angle measured before jack-knife stretching^11,14,15^. However, if the maximum knee extension angle measured after jack-knife stretching was smaller than that before the stretching, the muscle–tendon unit stiffness before and after the stretching was calculated from the 50% maximum knee extension angle to the maximum knee extension angle measured after the stretching^14,15^.

### Numerical rating scale

The level of pain during jack-knife stretching (Repetitions 1, 2, and 3) and post measurement was quantified by an 11-point NRS that ranged from 0 (no pain) to 10 (worst imaginable pain)^14,15^.

### Jack-knife stretching

Jack knife-stretching was performed in the same fashion as previous studies^19–21^ (Fig. 1). In the starting posture, participants squatted while holding their ankle joints with their hands. Subsequently, participants actively extended their knee joint to the target intensities (POD and PODmax intensity) while the chest and thighs remained in contact. This position is then held for 20 s. This procedure was repeated three times, with intervals of 30 s; three repetitions and two rest intervals. Two different intensities were performed based on the POD of each participant (POD and PODmax intensity). At POD intensity, the knee angle was set prior to the POD^14–16^. At PODmax intensity, the knee angle was set at the maximum angle that the participants tolerated, even if there was pain.

To measure the knee angle during jack-knife stretching, reflective markers were placed on the right greater trochanter, lateral condyle of femur, fibular head, and lateral malleolus. A video camera was used to capture an image of the sagittal plane during jack-knife stretching. The knee angle during jack-knife stretching was calculated from the images 10 s after the start of stretching by using Image J software (National Institutes of Health). The knee angle during jack-knife stretching was defined as the angle between the femur (the line passing through the greater trochanter and lateral condyle of femur) and the fibula (the line passing through the fibular head and lateral malleolus).

### Statistical analyses

The statistical analyses were performed according to previous studies^15,16^. All variables except NRS were described as mean ± SD in the present study. NRS was described as a median (interquartile range). A two-way repeated measures ANOVA (intervention [POD vs. PODmax] and time [PRE vs. POST]) was used to analyze the knee extension ROM, passive torque at end ROM, and muscle–tendon unit stiffness data. A two-way repeated-measures ANOVA (intervention [POD vs. PODmax] and time [first repetition vs. second repetition vs. third repetition]) was used to examine the knee angle during jack-knife stretching. A two-way repeated-measures ANOVA (intervention [POD vs. PODmax] and time [first repetition vs. second repetition vs. third repetition vs. post measurement]) was used to analyze NRS data. If a significance was detected, post hoc analyses using Bonferroni’s test were performed to determine where significant differences occurred. The analyses were performed using SPSS version 25 (SPSS, Inc., Chicago, IL, USA). Differences were considered statistically significant at an alpha level of p < 0.05. To describe the effect size, the partial eta squared value was calculated by using the SPSS software.

## Results

### Knee extension ROM

**Figure 2:**
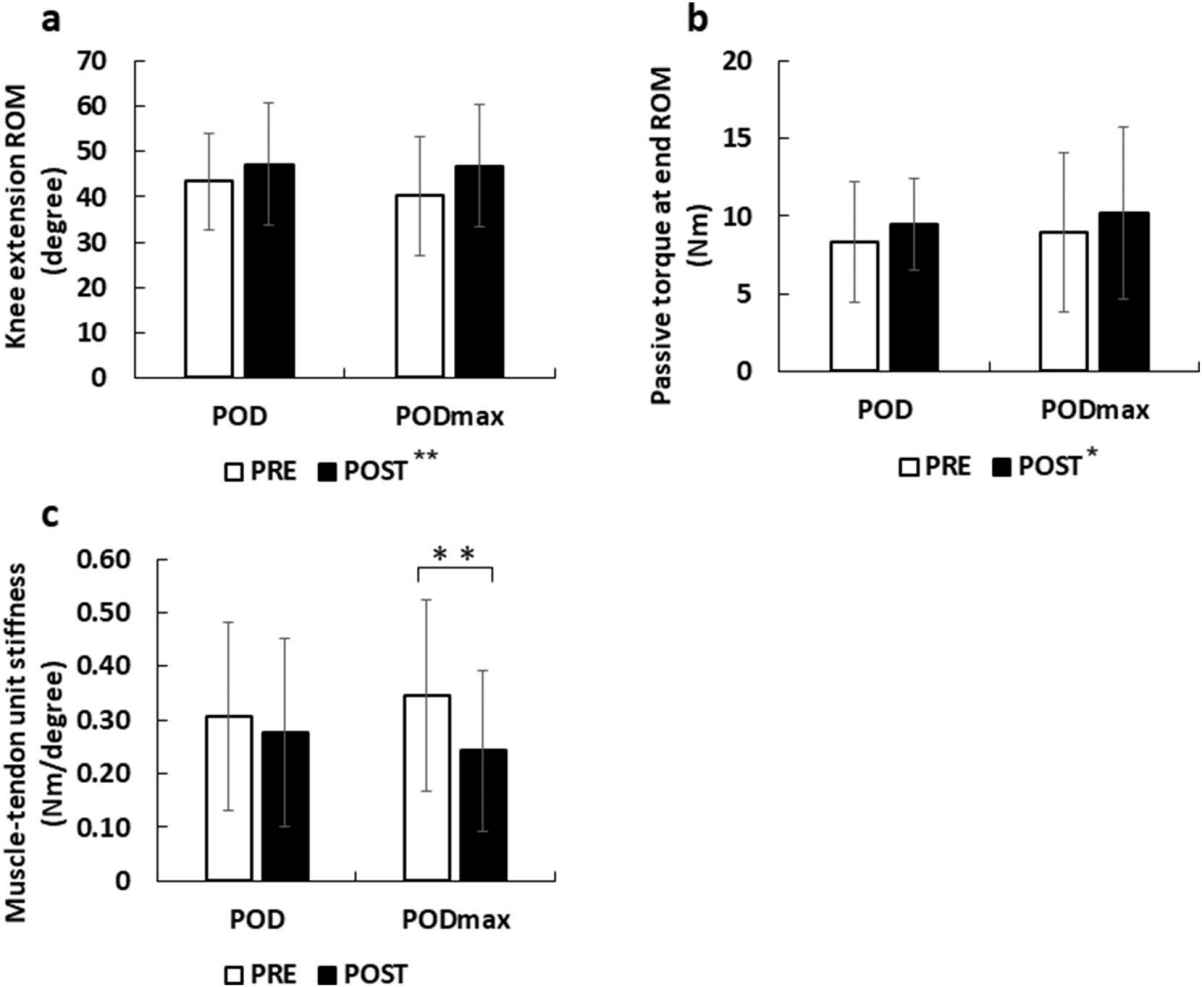
Changes in the knee extension range of motion (ROM) (**a**), passive torque at end ROM (**b**), and muscle–tendon unit stiffness (**c**). Data were represented as mean ± SD. *p < 0.05 (PRE vs. POST). **p < 0.01 (PRE vs. POST).

There was no significant interaction effect (p = 0.12, partial eta squared = 0.11) and no main effect for intervention (p = 0.73, partial eta squared = 0.01), but there was a significant main effect for time (p < 0.01, partial eta squared = 0.64) (Fig. 2). Post hoc analysis indicated the knee extension ROM at pre-stretching indicated significantly lower value compared to post-stretching (p < 0.01).

### Passive torque at end ROM

There was no significant interaction effect (p = 0.87, partial eta squared < 0.01) and no main effect for intervention (p = 0.68, partial eta squared = 0.01), but there was a significant main effect for time (p = 0.04, partial eta squared = 0.18) (Fig. 2). The passive torque at pre-stretching indicated significantly lower value compared to post-stretching (p = 0.04).

### Muscle–tendon unit stiffness

There was significant interaction effect (p = 0.03, partial eta squared = 0.24) (Fig. 2). The muscle–tendon unit stiffness significantly decreased in the PODmax (p < 0.01), while there was no change in the POD (p = 0.18).

### Knee angle during jack-knife stretching

There was no significant interaction effect (p = 0.73, partial eta squared = 0.01) and no main effect for time (p = 0.07, partial eta squared = 0.12), but there was a significant main effect for intervention (p = 0.01, partial eta squared = 0.26) (Table 1). The knee angle during jack-knife stretching at the POD indicated lower value compared to PODmax (p = 0.01).

**Table 1:** Knee angle during jack-knife stretching.

	First repetition	Second repetition	Third repetition
POD (°)	129.0 ± 16.1	124.0 ± 16.3	124.7 ± 20.4
PODmax* (°)	147.7 ± 19.3	137.6 ± 16.0	141.5 ± 13.9

Data were represented as mean ± SD.

*p < 0.05 (POD vs. PODmax).

### NRS

There was a significant two-way interaction (intensity × time, p < 0.01, partial eta squared = 0.56) (Table 2). In the first, second, and third repetitions, the median value of NRS of POD was significantly smaller than those of PODmax (all p < 0.01), while there was no significant difference between POD and PODmax in the post measurement (p = 0.23). At PODmax, NRS in the first, second and third repetitions were significantly higher values than those in the post measurement (all p < 0.01).

**Table 2:** NRS during jack-knife stretching and POST measurement.

	First repetition	Second repetition	Third repetition	Post measurement
POD	0 (0–3)^†^	0 (0–3)^†^	0 (0–3)^†^	0 (0–0)
PODmax	7 (6–8)*	6 (5–9)*	7 (5–9)*	0 (0–0)

Data were represented as median (interquartile range).

*p < 0.01 vs. values in post measurement at PODmax.

^†^p < 0.01 vs. values at PODmax in the same stretching repetition.

## Discussion

In the present study, the effects of different intensities (POD and PODmax) of jack-knife stretching on the knee extension ROM, passive torque at end ROM, muscle–tendon unit stiffness, knee angle during the stretching, and NRS were examined. The results showed that the knee extension ROM and passive torque at end ROM increased regardless of its intensity, although the muscle–tendon unit stiffness decreased only after jack-knife stretching at the intensity of PODmax. Although there have been some studies that have investigated the effects of passive high-intensity static stretching of the hamstrings^14–16^, this is the first paper to investigate the effect of high-intensity self-stretching on the muscle–tendon unit stiffness.

In the present study, the knee angle during jack-knife stretching was 10°–20° greater in PODmax than that in POD. In the PODmax intensity, the median values of NRS during jack-knife stretching were high (levels 6–7) although the pain disappeared post measurement, which results consistent with previous studies^15,16^. In previous studies, high-intensity static stretching was performed in a posture where the knee extension angle during the high-intensity static stretching was approximately 10°–20° greater than the static stretching at the intensity of POD^14–16^. Besides, it was reported that NRS during the passive high-intensity static stretching was 5–9 but the pain disappeared after stretching and 24 h after interventions^14–16^. Taken together, it indicated that the intensity of jack-knife stretching at the intensity of PODmax used in this study was the same intensity of high-intensity static stretching in the previous studies^14–16^. Moreover, it was indicated that high-intensity jack-knife stretching may be as safe as high-intensity static stretching.

In the present study, the knee extension ROM and passive torque at end ROM increased but the muscle–tendon unit stiffness was not changed after 60 s of jack-knife stretching at the intensity of POD. The change in ROM after static stretching was attributed to alterations in stretching tolerance^25–28^ and muscle–tendon unit stiffness^11,29–31^. In the present study, stretching tolerance was examined by using passive torque at end ROM^25–28^. Matsuo et al.^11^ and Nakamura et al.^29^ reported that more than 180 s of static stretching at the intensity of POD was needed to decrease the muscle–tendon unit stiffness. To our best knowledge, changes in stretching tolerance and muscle–tendon unit stiffness after jack-knife stretching have not been investigated. The results of the present study indicated that 60 s of jack-knife stretching at the intensity of POD increased the knee extension ROM through increasing stretching tolerance without changing the muscle–tendon unit stiffness.

In the present study, the knee extension ROM and passive torque at end ROM increased but the muscle–tendon unit stiffness decreased after 60 s of jack-knife stretching at the intensity of PODmax. Kataura et al.^14^ compared the effects of 180 s of different intensities of static stretching (80, 100, and 120%ROM) on the muscle–tendon unit stiffness of the hamstrings. They reported that high-intensity static stretching (120%ROM) was most effective for the decrement in the stiffness. Similarly, Takeuchi and Nakamura^15^ reported that 20 s of high-intensity static stretching at an intensity of 120%POD or more significantly decreased the muscle–tendon unit stiffness of the hamstrings. Moreover, Takeuchi and Nakamura^16^ reported that high-intensity static stretching was effective for the decrement in the muscle–tendon unit stiffness of the hamstrings even if with a short stretching duration (≤ 20 s). Taken together, it was suggested that high-intensity jack-knife stretching decreased the muscle–tendon unit stiffness in a short time (≤ 60 s), as has been previously found with passive high-intensity static stretching^15,16^. Recent studies showed that the intensity of static stretching may be more important than its duration to decrease the muscle–tendon unit stiffness of the hamstrings^16^ and the muscle stiffness of medial gastrocnemius^17^. However, the mechanisms of the decrement in muscle stiffness after high-intensity static stretching are unclear.

There were some limitations in this study. Firstly, the present study examined high-intensity self-stretching on the muscle–tendon unit stiffness of the hamstrings. Previous studies showed that alteration in the muscle–tendon unit stiffness was correlated with the intensity of static stretching in the hamstrings^15,16^. However, Nakamura et al.^18^ reported that high-intensity static stretching (120%ROM) was not effective for a decrement in muscle stiffness of the rectus femoris because it is overly stressful. These data indicated that the effects of high-intensity static stretching may differ depending on the targeted muscles. Therefore, the effects of high-intensity self-stretching need to be examined in muscles other than the hamstrings. Secondly, the hamstrings are composed of three muscles (semitendinosus, semimembranosus, and biceps femoris muscles). In these three muscles, muscle strain most often occurs in the biceps femoris^32–34^. However, the results of the present study did not show which muscle was affected by high-intensity jack-knife stretching. It is necessary to examine the changes in the muscle stiffness of each muscle using shear wave elastography. Thirdly, the present study examined only the acute effects of high-intensity jack-knife stretching. Therefore, the long-term effects of high-intensity jack-knife stretching or its effectiveness in preventing muscle strain remain unclear. Further research is planned to elucidate long-term effects, using a long-term intervention study. Finally, previous studies showed that gender difference may affect the changes in flexibility after static stretching at the intensity of POD^22,35^. Therefore, any gender differences in high-intensity stretching also need to be examined.

In conclusion, jack-knife stretching at the intensity of PODmax increased the knee extension ROM and passive torque at end ROM and decreased the muscle–tendon unit stiffness of the hamstrings. The pain during high-intensity jack-knife stretching disappeared after the stretching. These results suggested high-intensity jack-knife stretching is an effective and safe method to decrease muscle–tendon unit stiffness of the hamstrings.

## Data Availability

The datasets generated during and/or analyzed during the current study are available from the corresponding author on reasonable request.
